# There is no well-being without oral health: a case report of Lemierre’s syndrome due to neglected odontogenic infection

**DOI:** 10.1590/1677-5449.202301622

**Published:** 2024-08-09

**Authors:** Edward Davis, Ardeno Kristianto, Andrew Jackson Yang

**Affiliations:** 1 Atma Jaya Catholic University of Indonesia, Jakarta, Indonesia.; 2 St. Carolus Hospital, Jakarta, Indonesia.

**Keywords:** Lemierre’s syndrome, septic thrombophlebitis, dental infection, anticoagulant, síndrome de Lemierre, tromboflebite séptica, infecção dentária, anticoagulante

## Abstract

Lemierre’s syndrome is marked by presence of septic thrombophlebitis in the internal jugular vein. This case report describes a 57-year-old woman who presented with a progressively swelling neck with onset 1 day prior to admission. She had a history of untreated dental infection. Physical examination revealed slightly increased blood pressure, at 140/80 mmHg, and a painful, erythematous, warm swelling in the mid area of the neck. Ultrasound of the neck revealed occlusive intraluminal thrombus in the right internal jugular vein, a computed tomography (CT) scan with contrast showed that there was a blockage in the right jugular vein. The mainstay treatment for Lemierre’s syndrome is antibiotics, while administration of anticoagulants remains controversial. The patient was treated conservatively, with administration of antibiotics and anticoagulant. Several days later the patient’s condition had improved significantly, with less pain and reduced swelling.

## Resumo

A síndrome de Lemierre é caracterizada pela presença de tromboflebite séptica da veia jugular interna. Este relato de caso descreve uma mulher de 57 anos que apresentou edema progressivo no pescoço 1 dia antes da internação. Ela tinha histórico de infecção dentária não tratada. O exame físico revelou pressão arterial levemente aumentada (140/80 mmHg), além de protuberância dolorosa, eritematosa e com aumento da temperatura na região média do pescoço. A ultrassonografia do pescoço revelou trombo intraluminal na veia jugular interna direita, e a tomografia computadorizada com contraste mostrou que havia trombose na veia jugular direita. O tratamento principal da síndrome de Lemierre é a antibioticoterapia, enquanto a administração de anticoagulantes permanece controversa. A paciente foi tratada de forma conservadora com administração de antibióticos e anticoagulantes. Vários dias depois, a condição da paciente melhorou significativamente, com diminuição do nível de dor e do tamanho da protuberância.

## INTRODUCTION

Lemierre’s syndrome (LS) was first described by Courmont and Cade in 1900; however it was only named Lemierre’s syndrome in 1936 by a French microbiologist Andre Lemierre.^1^ It is characterized by bacteremia due to oropharyngeal infection with thrombophlebitis in the internal jugular vein.^2^ Fusobacterium necrophorum, an anaerobic gram negative bacilli, is commonly responsible for this condition,^3^ while other pathogens that can also rarely cause this condition include *staphylococci sp, klebsiella sp*, and *streptococci sp*.^4^ LS is rare but potentially fatal and its incidence has decreased since the introduction of antibiotics. It is estimated that the incidence of LS is between 0.6 and 2.3 million cases with a mortality rate as high as 18%.^5^ Antibiotics are the mainstay treatment for LS, while surgical procedures are rarely needed.^6^ In this case report, we describe a 57-year-old female who developed LS due to an untreated odontogenic infection.

Ethics committee clearance was obtained for this study (019/SB/KEP-RSSC/LOLOS UJI ETIK/XII/2023). This study complies with the Helsinki Declaration and local ethical guidelines. Written informed consent was obtained from the patient.

## CASE PRESENTATION

A 57-year-old female was admitted to the emergency department on 16th September 2022 with a swollen neck with onset 1 day before admission. The patient described the swollen neck as painful and enlarging progressively since 1 day prior. The patient had history of cough since 1 month prior; however she reported that there had been no episodes of fever. She had a history of caries and tooth radix which were left untreated. There was no history of sudden weight loss or swelling in other parts of the body.

Upon physical examination, her blood pressure was slightly elevated (140/80 mmHg), with heart rate 89 bpm, respiratory rate 20 bpm, temperature 36.8 C, and oxygen saturation 98% on room air. Neck examination showed swelling in the mid area of the neck. The swelling was erythematous and warm upon palpation. Chest examination revealed vesicular sound in all regions of the lung, cardiac sounds were normal, and there was no gallop or murmur.

**Figure 1:**
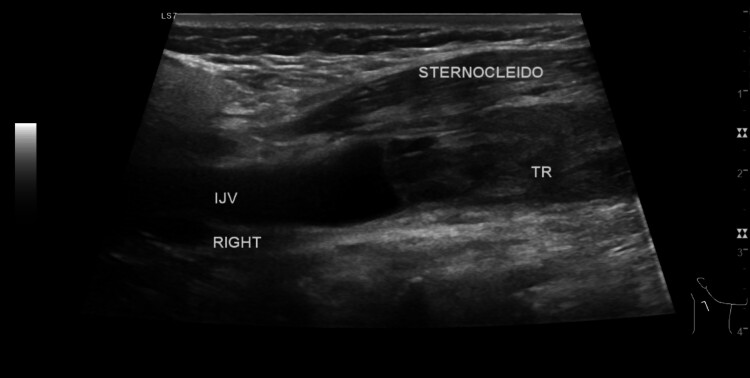
Neck ultrasound revealed intraluminal thrombus in the right internal jugular vein.

**Figure 2:**
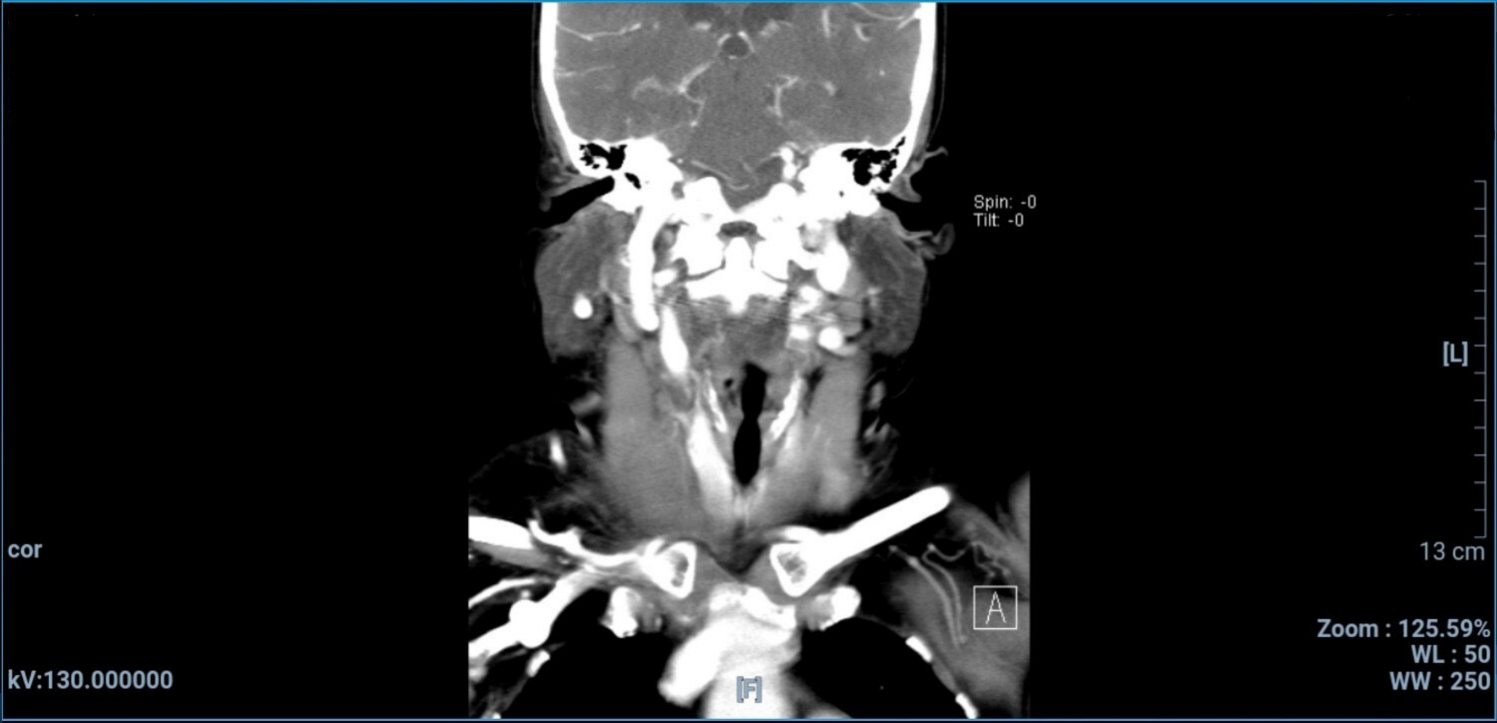
Coronal view of CT scan with contrast showing filling blockage in the right jugular vein.

**Figure 3:**
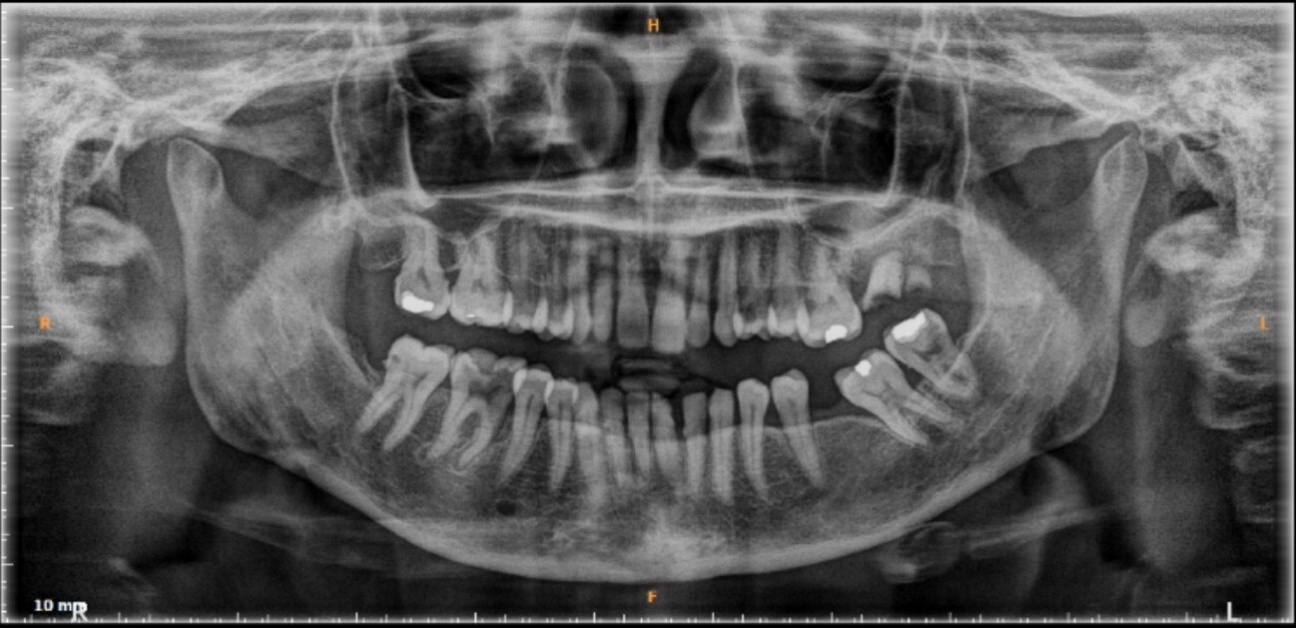
Panoramic X-ray revealed multiple missing teeth, teeth radix, tooth extrusion, and tooth with periapical lucency.

Initially, a neck abscess was suspected as the cause of the swelling. Neck ultrasound was performed and revealed intraluminal thrombus with total occlusion of the right internal jugular vein, fat stranding, and a reactive lymph node in nearby tissue (Figure 1). There was no abscess formation or any abnormality in the thyroid, submandibular gland, or parotid gland. Laboratory examination on 16th September 2022 showed leukocytosis at 13.38 x 10^3^/ μL and an increased erythrocyte sedimentation rate at 84 mm. On 17th September 2022, a neck CT scan with contrast showed that right jugular vein filling was blocked, suggesting phlebitis accompanied by thrombus in the right jugular vein (Figure 2). Panoramic X- ray was also performed afterwards to evaluate dental condition, finding that multiple teeth were missing and showing teeth radix, tooth extrusion, and a tooth with periapical lucency (Figure 3).

The patient was treated with anticoagulants, antibiotics, and non-steroidal anti-inflammatory drugs (NSAID). On 16th September 2022, heparin was administered, 4000 units via intravenous bolus, and continued with 20,000 unit/24 hr. Levofloxacin 750 mg qd and metronidazole 500 mg tid were administered intravenously. Ketorolac 30 mg tid was also given intravenously to alleviate the pain and inflammation. The patient’s condition improved rapidly afterwards and on 19th September the patient stated she had no more pain in the neck and the size of the swelling had also reduced. The patient was discharged on 21st September 2022 and was treated in outpatients. Heparin was replaced with warfarin 2 mg per oral qd. Levofloxacin 750 mg qd was continued orally for 5 days and oral metronidazole 500 mg tid was continued for 25 days. Subsequently, the patient was referred to a dental clinic to get root canal treatment and teeth radix extraction.

**Figure 4:**
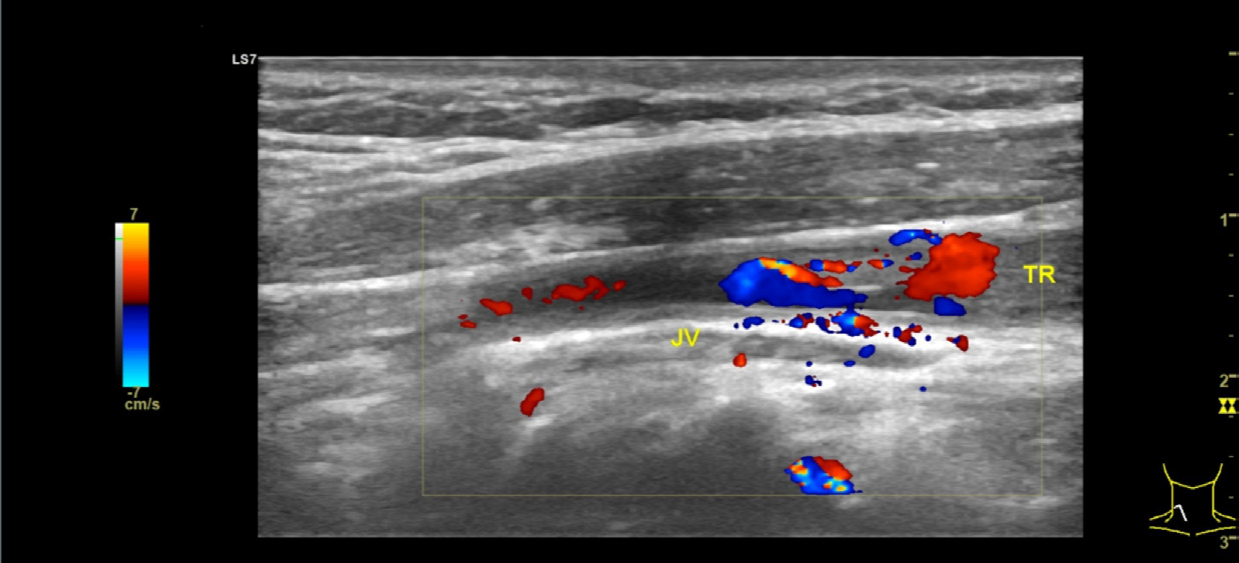
Follow up neck ultrasound revealed partially restored blood flow.

Neck ultrasound was repeated on 29th September 2022 and revealed that reactive lymph node and fat stranding were no longer visible, but intraluminal thrombus was still visible in the right jugular vein. On 5th October 2022, warfarin was replaced with high dose rivaroxaban 15 mg bid. Rivaroxaban was tapered down on 19th October 2022 to 20 mg qd. Subsequent neck ultrasound on 7th November 2022 showed that blood flow was visible posterior to the thrombus (Figure 4).

## DISCUSSION

LS is traditionally diagnosed with diagnostic criteria that include: (1) history of oropharyngeal infection, (2) thrombophlebitis in the internal jugular vein, (3) septic emboli in remote site, and (4) the presence of *fusobacterium sp* from blood culture. Currently it is proposed that the most accurate definition of Lemierre’s syndrome would be bacteremia due to ear-nose-throat (ENT) infection that causes thrombophlebitis in the internal jugular vein and produces metastatic emboli in the periphery.^7^ Our patient presented with a painful neck swelling and cough and had a history of untreated odontogenic infection. Common symptoms of LS include fever (82.5%-100%), sore throat (82.5%), swollen and/or tender neck (52.2%), and jaundice (33%).^8^ As found in other cases, the symptoms in our patient may not look severe; however this should not reduce our suspicion since diagnosis of LS is mainly clinical.^9^

Bacterial pathogens might be identifiable in blood cultures, but it is not rare to obtain negative culture results. The absence of pathogens in blood culture should not negate a diagnosis of LS.^10^ Various imaging modalities, such as CT scan, simple echography, or duplex scan in the cervical region can be used to establish definitive diagnosis of LS.^11^ CT scan with contrast has the highest diagnostic value, since it will reveal soft tissue edema and filling failure, and it might even show the thrombus in the internal jugular vein.^11,12^

Intravenous antibiotics is the first line treatment for LS. Antimicrobial therapy should target anaerobic bacteria. Fusobacterium necrophorum, which is the most common etiologic agent, has high sensitivity to penicillin, clindamycin, and metronidazole.^13^ Use of anticoagulant in LS is still controversial.^14^ However, in our case we found that administration of anticoagulant was able to prevent the extension of thrombus in the internal jugular vein after approximately 6 weeks of anticoagulation. Surgical intervention in the form of internal jugular vein resection is rarely needed, nonetheless it can be indicated should there be persistent septic emboli despite pharmacological therapy.^11^

## CONCLUSION

Lemierre syndrome is rare nowadays; but it is still present. It is important for clinicians to be able to recognize this condition early, since it can potentially be fatal if left undiagnosed and untreated.
